# Properties of 3D-Printed resins for interim restorations: effects of printing and post-curing protocols

**DOI:** 10.1590/1807-3107bor-2025.vol39.019

**Published:** 2025-02-21

**Authors:** Leandro Edgar PACHECO, Fernanda Balestrero CASSIANO, Vitor de Toledo STUANI, Isabela Sanches Pompeo da SILVA, Larissa ALAMO, Matheus de Castro COSTA, Marcella Fernandes LOVISON, Sergio Kyioshi ISHIKIRIAMA, Rafael Francisco Lia MONDELLI, Diana Gabriela SOARES

**Affiliations:** (a) Universidade de São Paulo – USP, Bauru School of Dentistry, Department of Operative Dentistry, Endodontics and Dental Materials, Bauru, SP, Brazil.

**Keywords:** Acrylic Resins, Printing, Three-Dimensional

## Abstract

This study analyzed the properties of three-dimensional (3D)-printed resins designed for interim restorations and subjected to different printing and post-curing protocols. Specimens were manufactured with two 3D-printed resins for interim restorations at varying exposure times per layer, in accordance with the manufacturer’s recommendations (Tm) or using a calibrator (Tc). Subsequently, the specimens were post-cured for 5, 10, or 15 min. Trueness, surface roughness, and color analyses were performed. For biological characterization, the specimens were incubated for up to 72 h in a culture medium, and the extracts were applied at 24-h intervals to keratinocyte cells (NOK-Si). Cellular metabolism was evaluated after 1 and 3 days. Leaching of residual monomers from the extracts was evaluated. Data were analyzed using two-way analysis of variance (ANOVA) and Tukey’s honestly significant difference (HSD) test (α = 5%). The exposure time beyond Tc resulted in specimens with increased trueness and smoother surfaces. Color stability was also influenced by the type of resin and post-curing time; the longer the time, the greater the color change, allowing for brighter and clearer specimens. The resins were cytocompatible with NOK-Si, regardless of the printing and post-curing parameters, although residual monomer leaching was affected by the parameters tested. The evaluated resins were cytocompatible; however, variations in the exposure times per layer and post-curing duration affected their roughness, leaching, trueness, and color stability.

## Introduction

Intraoral scanning and three-dimensional (3D)-printing technologies have accelerated the adoption of digital workflows in clinical dental practice, making computer-based fabrication methods for dental restorations an increasingly promising field.^1,2^ The use of 3D-printed resins in dentistry has resulted in the introduction of a new category of materials, with several advantages, including versatility, replicability, low cost, practicality, reduced chair time, adequate mechanical properties, and good precision.^3-7^ Consequently, interest in the use of additive manufacturing has increased recently.^3^ However, the parameters that define the efficiency of the 3D-printing process vary based on the printing technology, method, and material.^8^ These parameters can affect the accuracy, trueness, mechanical properties, surface characteristics, color stability, and attachment of microorganisms to 3D-printed materials.^3^

Another important aspect is the post-printing protocol, more specifically the post-polymerization performed using ultraviolet (UV) light.^8^ This protocol contributes to the conversion of residual unreacted monomers in the outer layers.^7-10^Despite the guidelines provided by manufacturers of resin and 3D printers, limited scientific information is available regarding the best printing process and post-curing techniques required to achieve restorations with adequate mechanical and aesthetic properties from 3D-printed resins.^11^Information on the chemical composition and biological parameters of these materials is also scarce.^4,7,8,10^ Since the biocompatibility of provisional resin restorations with periodontal tissue cells is directly related to the leaching of residual unreacted monomers,^7,12^ the effects of printing and post-printing parameters on the cytocompatibility of oral tissues is an important consideration.^6^

In terms of aesthetic performance, 3D-printed resins show high variability in shades^11^ and poor color stability after water storage.^2,11^ Moreover, prolonged exposure to violet light from the post-curing unit can affect the color of the final restoration.^11,13-15^ Another important factor to assess is the surface smoothness of provisional restorations, since this is essential for preventing biofilm accumulation and maintaining the health of periodontal tissues.^2^Therefore, this study aimed to evaluate the influence of factors related to the manufacturing process of interim restorations using additive technology (3D printing) on the trueness, surface roughness, color stability, residual monomer leaching, and cytotoxicity to oral mucosal cells. The null hypothesis was that the layer exposure and post-curing time have no effects on the parameters tested in the present study.

## Methods

### Experimental groups

Two 3D-printed resins were evaluated: PZP (Prizma Bio Prov DLP/LCD A1; Makertech Labs, Tatuí, SP, Brazil) and SPP (Smart Print Bio Temp A1; Smart Dent, São Carlos, SP, Brazil). Two parameters were varied when processing the resins: a) exposure time per layer – the time recommended by the manufacturer (Tm) of the printer used and the time determined by a calibrator (Tc); b) post-curing time - the specimens were post-cured for periods of 5, 10, and 15 min in a UV light chamber. Conventional chemically activated acrylic resin (Dencôr Color 61; Artigos Odontológicos Clássico Ltda, São Paulo, Brazil) was used as the control. The material was manipulated according to the manufacturer’s recommendations and inserted into a metallic matrix (14 mm diameter × 1 mm thickness) between two glass slides covered with a thin layer of Vaseline to avoid the oxygen inhibition layer. This set of conditions was maintained under controlled pressure during polymerization at room temperature (24^°^C). No surface polishing was performed on the specimens. The experimental groups are listed in Table.

**Table:** Experimental groups respective to the layer exposure time, pos-curing time and composition.

Group	Resin	Layer exposition time	Post-curing time	Composition
PZP	Prizma Bio Prov A1 DLP/LCD	2.5 s	5 min.	Oligomers, Monomers, Photoinitiators, Stabilizer, Pigment
Tm/5min				
PZP			10 min.	
Tm/10min				
PZP			15 min.	
Tm/15min				
SPP	Smart Print Bio Temp B1 DLP/LCD	1.8 s	5 min.	Oligomers, Monomers, Photoinitiators, Stabilizer, Pigment
Tm/5min				
SPP			10 min.	
Tm/10min				
SPP			15 min.	
Tm/15min				
PZP	Prizma Bio Prov A1 DLP/LCD	5.0 s	5 min.	Oligomers, Monomers, Photoinitiators, Stabilizer, Pigment
Tc/5min				
PZP			10 min.	
Tc/10min				
PZP			15 min.	
Tc/15min				
SPP	Smart Print Bio Temp B1 DLP/LCD	2.0 s	5 min.	Oligomers, Monomers, Photoinitiators, Stabilizer, Pigment
Tc/5min				
SPP			10 min.	
Tc/10min				
SPP			15 min.	
Tc/15min				
Acrylic Resin	Chemically activated acrylic resin	-	-	Powder: methyl methacrylate copolymer. Liquid: methyl methacrylate monomer

### 3D-printing parameters

**Figure 1:**
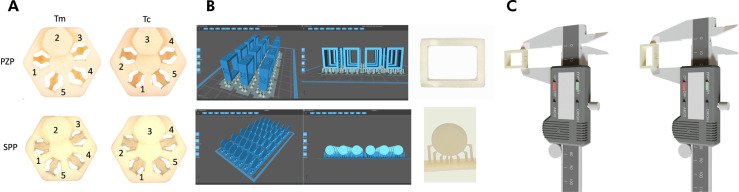
(a) Representative images of calibration tool for Tc selection. Numbers are representative of holes of calibrator. Number 3 is representative of ideal adaptation for which Tc was selected. (b) Representative images of CAD design of specimens on printing bed in slicer software and printed specimens (rectangular and cylindrical). (c) Schematic representation of trueness analysis of x- and z-axis.

For the 3D-printed resins, a calibrator with a male–female fitting (Makertech Labs) was used to determine the Tc. This calibrator consisted of two structures: a hexagonal base with five smaller hexagonal holes numbered 1 to 5 and a pivot consisting of a disc-shaped apex and a hexagonal pin. The layer exposure time parameter was tested until the pivot fitted precisely into the corresponding hole number 3 in the base, which represented the dimensions of the computed-aided design (CAD) project. For both the PZP and SPP resins used in this study, the layer exposure time was adjusted such that the calibrator structures fit properly, as shown in Figure 1a.

### Preparation of 3D-printed specimens

Specimens in the form of hollow rectangles (x-axis = 15 mm, z-axis = 20 mm) were obtained for trueness analysis. Disc-shaped specimens (Ø 14 mm × 1 mm thick) were obtained for the cell viability test, residual monomer analysis, surface roughness, and color stability assessments. The specimens were designed using open-access CAD software (Meshmixer; Autodesk ®, Inc., San Rafael, USA) and printed on a 3D printer (Anycubic Photon Mono SE - SLA/LCD; Anycubic, Shenzhen, China) according to the respective parameters. The specimens were then washed with isopropyl alcohol for 2 min and post-cured in a UV light chamber for different periods (Wash and Cure 2.0; Anycubic, Shenzhen, China). For all impressions, the layer thickness was standardized at 50 μm, and the positioning of the piece on the table was 90 degrees.^6^ Figure 1b demonstrates the CAD design placed onto the printing bed on slicer software (Chitubox Pro V1.3.0) and printed specimens.

### Trueness analysis

Trueness assessments were based on a previous study,^16^ in which the specimens followed the design of an openly available model in the form of a hollow rectangle (n = 10) (Figure 1b). The 3D-printing calibration model was chosen to evaluate the printing distortion in the x- and z-axes, and these dimensions were measured with a digital caliper (Model HDCD01150; INGCO, Lençóis Paulista, Brazil) by the same operator, with three measurements taken in each axis (Figure 1c). The data were obtained by subtracting 15 mm (x-axis) and 20 mm (z-axis) from the measured value of each specimen; thus, the results represented the difference in millimeters.^17^The data were also normalized by division, with the measured values of the specimens divided by 15 (x-axis) and 20 (z-axis), showing how close the groups were from the ideal standard (threshold = 1).

### Surface roughness

This analysis was performed using a bench roughness meter (Hommel TesterT1000; JenoptikAG, Jena, Germany) immediately after the specimens were obtained (Table). Acrylic resin specimens were used as controls. The specimens were stabilized with a silicone support, and measurements were performed five times per sample (n = 4). The circular specimens were divided into two perpendicular axes at the center (vertical and horizontal). Measurements were performed three times parallel to the vertical line and twice parallel to the horizontal line. The measurements were performed with a reading accuracy of 0.01 µm, a reading length of 2.5 mm, and an active tip speed of 0.5 mm/s. Surface roughness (Ra) values were obtained from the arithmetic means of the readings.

### Color analysis

This analysis was performed solely for 3D-printed specimens (Table) to evaluate the effects of the 3D-printing and post-curing parameters. A portable spectrophotometer (Easy Shade Advanced 4.0; VITA Zahnfabrik, Bad Säckingen, Germany) was used, and the spectral distribution of color was calculated based on the CIELab (ΔE*_ab_) and CIEDE2000 (ΔE_00_) equations. Both color systems are based on three main parameters: L* refers to the luminosity (L* = 0 = black; L* = 100 = white), a* indicates the chroma on the red-green axis (a* > 0 = red and a* < 0 = green), and b* is the chroma on the yellow-blue axis (b* > 0 = yellow; b* < 0 = blue).

A white silicone device was used to stabilize the specimens, and three standardized measurements (n = 6) were performed at the top of the specimens. The color changes at different post-curing times were measured by calculating the variations in L* (ΔL), a* (Δa), and b* (Δb) {0 (no post-curing), 5, 10, and 15 minutes}.

E *_ab_ was calculated using the following equation:


$${\Delta E}_{ab}^{*}\;=\;[{\left(\Delta L^{*}\right)}^{2}\;+\;{\left(\Delta a^{*}\right)}^{2}\;+\;{\left(\Delta b^{*}\right)}^{2}{]}^{1/2}$$

For CIEDE2000, ΔL′, AC′, and AH′ represent luminosity, chroma, and hue, respectively. ΔR = RT (ΔC′ × ΔH′) refers to the interaction between chroma and hue in the blue region. SL, SC, and SH are weighting functions that adjust the total color difference in the L*, a*, and b* coordinates, respectively. KL, KC, and KH are the parametric factors that serve as correction terms for the experimental conditions.

ΔE_00_ was calculated using the following equation:


$${\Delta E}_{00}\;=\left[\;{\left(\frac{\Delta L'}{K_{L}S_{L}}\right)}^{2}\;+\;{\left(\frac{\Delta C'}{K_{C}S_{C}}\right)}^{2}\;+\;{\left(\frac{\Delta H'}{K_{H}S_{H}}\right)}^{2}\;+\;RT\;\left(\frac{\Delta C'}{K_{C}S_{C}}\right)\;{\left(\frac{\Delta H'}{K_{H}S_{H}}\right)}^{1/2}\right]$$

### Cell cytotoxicity

An in vitro test was performed using spontaneously immortalized oral keratinocytes (NOK-Si; RRID:CVCL_BW57). This cell lineage was registered by the International Cell Line Authentication Committee; therefore, Ethical Committee approval was not required to perform the biological experiments. A monolayer of NOK-Si cells was seeded (5 × 10^4^ cells) in the wells of 96-compartment plates in Dulbecco’s modified Eagle’s medium (DMEM) supplemented with 10% fetal bovine serum (FBS), l-glutamine, and 1% penicillin-streptomycin (Gibco, Invitrogen) for 24 h. The acrylic resin and 3D-printed resin specimens (Table) were sterilized under UV-C light (254 nm; 15 W) for 30 min on each side, at a distance of 610 cm and room temperature (24^o^C), inside the laminar flow hood (Filterflux, Mod SBIIA1-960/4, São Carlos, SP, Brasil). The sterilization process had no curing effect on the specimens owing to the distance between the specimens and the UVC lamp. The effect of curing on residual monomer leaching was tested in a pilot study to ensure its applicability in the present investigation (data not shown). Thereafter, specimens were immersed in 1 mL of the culture medium and incubated (37°C/5% CO_2_) in 24-compartment plates (extraction rate of 3 cm^2^ /mL) for up to 72 h, as recommended in ISO10993-5:2009.^18^ In the negative control group, only the medium without specimens was incubated on the plates. During the experiment, the extract was applied to the cells every 24 h (continuous exposure model). The (3-(4,5-dimethylthiazol-2yl)-2,5-di-phenyl tetrazolium bromide (MTT) assay (Invitrogen) was performed after 1 and 3 days of culture (n = 8) to measure cell metabolic activity as an indicator of cell viability. The formazan crystals were dissolved in acidified isopropanol (Sigma-Aldrich, San Luis, USA), and the absorbance was measured at 570 nm (Synergy H1; Biotek, Winooski, USA). The mean absorbance values of the negative control group at each period were considered to separately reflect 100% cell viability for that period.

### Residual monomer leaching

This analysis was performed on the 24-h extracts used in the cell viability assay. The absorbance was measured at 270 nm (Synergy H1; Biotek, Winooski, VT, USA), as determined by Alamo et al.^6^ The plain culture medium from the negative control group was used as blank.

### Statistical analysis

The sample size for quantitative assays was calculated using DDS Research (Sample Size Calculator; average, 2 specimens; α = .05; β = .95). Two independent experiments were performed. Data were compiled and analyzed using the Shapiro-Wilk test (p > 0.05) and Levene’s test (p > 0.05). One-way ANOVA was used to detect significant effects of the experimental group at each exposure time. Tukey HSD test was used for observation of the significant differences between the study groups at each exposure time (p < 0.05). Dunnett’s test was used to observe significant differences between the study groups and the standard for trueness analysis (p < 0.05).

## Results

For all analyses, the resins were divided into two subgroups based on the exposure time (Tm and Tc).

### Trueness

**Figure 2:**
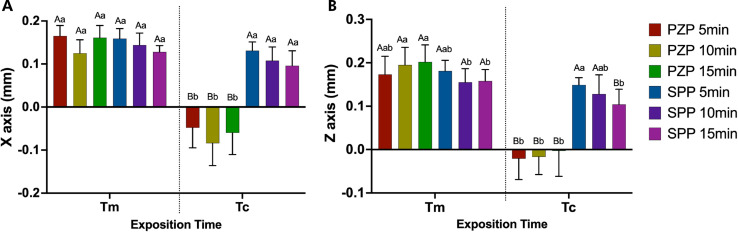
Trueness analysis in millimeters for x-axis (a) and z-axis (b) (data obtained by subtraction). Bars represent mean and standard deviation.

**Figure 3:**
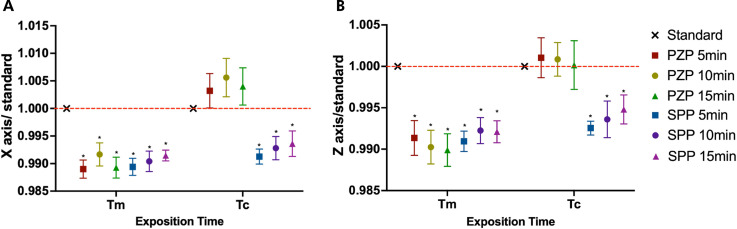
Trueness analysis x-axis (a) and z-axis (b) related to an ideal threshold (data obtained by division).

The trueness results in millimeters for the x- and z-axes are shown in Figure 2. On the x-axis, the discrepancy between the values was smaller when Tc was used. However, a significant change was observed only for the PZP resin when comparing the Tm and Tc subgroups (p < 0.001), regardless of the post-curing time. A similar result was observed for the z-axis: the PZP resin showed a considerable reduction in the discrepancy between the Tc and Tm subgroups (p < 0.001), regardless of the post-curing time. In contrast, the SPP resin showed a significant reduction only for the 15-min post-curing time in the z-axis (p < 0.01). Figure 3 shows the normalized data by axes (x and z). On both axes, the values were closer to the ideal for Tc, with only the PZP resin showing no significant difference from the threshold (standard line) at any post-curing time (p > 0.05).

Capital letters allow comparison between Tm and Tc for each group. Lowercase letters allow comparison between groups at each exposition time. Different letters indicate statistically significant differences (One-way ANOVA/ Tukey’s test. p < 0.05).

Boxes represent mean and standard deviation. Asterisks indicate significant differences with standard (One-way ANOVA/ Dunnet’s test. p < 0.05).

### Surface roughness

**Figure 4:**
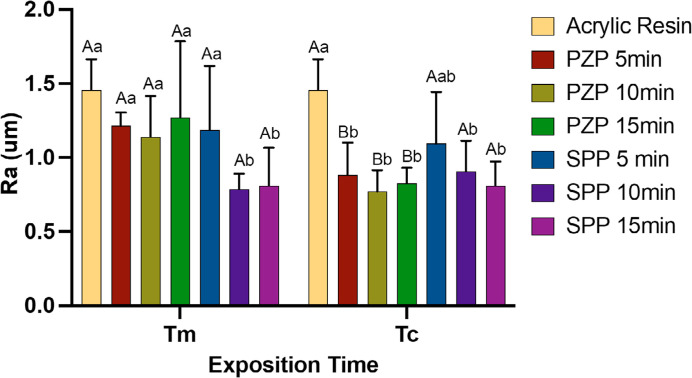
Roughness analysis. Bars represent mean and standard deviation.

Significant differences were observed among the materials tested (p < 0.01) for each exposure time per layer (Figure 4). The acrylic resin showed the highest surface roughness values. For the PZP resin, the surface roughness was statistically similar to that of the acrylic resin for Tm (p > 0.05); however, a significant reduction in roughness was observed for Tc (p < 0.001), which was significantly lower than that of the acrylic resin (p < 0.05). The post-curing time did not affect the results. Significantly lower roughness values were observed for the SPP resin than for the acrylic resin (p < 0.01) when the resins were subjected to post-curing times of 10 and 15 min, regardless of the exposure time per layer.

Capital letters allow comparison between Tm and Tc for each group. Lowercase letters allow comparison between groups at each exposition time. Different letters indicate statistically significant differences (One-way ANOVA/ Tukey’s test. p < 0.05).

### Color analysis

**Figure 5:**
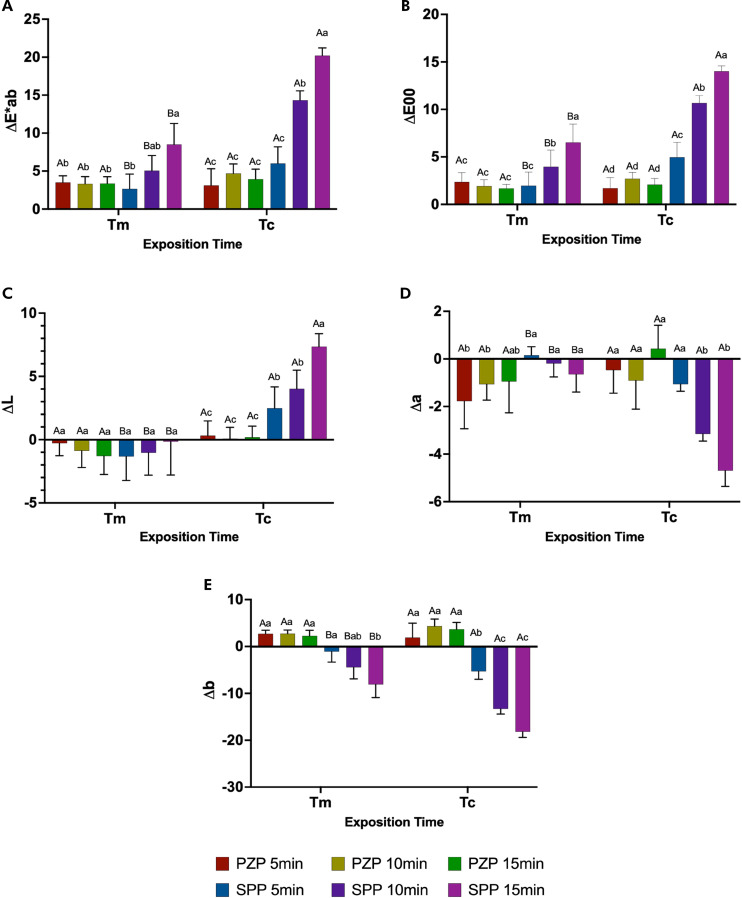
Color analysis. Bars represent mean and standard deviation for (a) ΔE*ab (b) ΔE00, (c) ΔL, (d) Δa, and (e) Δb.

The results for the ΔE*_ab_, ΔE_00_ ΔL, Δa, and Δb variables are depicted in Figure 5. For the total color variation (ΔE*_ab_ and ΔE_00_), the post-curing time had a greater influence in the SPP group. ΔE*_ab_ showed a significant increase with post-curing at 15 min for Tm (p < 0.05) and at 10 and 15 min for Tc (p < 0.01). For ΔE_00_, the effect of post-curing was more evident, with significant increments observed at 10 and 15 min for Tm (p < 0.01) and at 5, 10, and 15 min for Tc (p < 0.001). A significant difference in ΔE*_ab_ (p < 0.05) and ΔE_00_ (p < 0.001) values was observed for the SPP resin at all post-curing times in comparison with the exposure times per layer. No differences were found for PZP, regardless of layer exposure and post-curing time (p > 0.05).

Capital letters allow comparison between Tm and Tc for each group. Lowercase letters allow comparison between groups at each exposition time. Different letters indicate statistically significant differences (One-way ANOVA/ Tukey’s test. p < 0.05)

The influence of each variable on the total color variation can be assessed by analyzing each color parameter individually. For ΔL (color luminosity), the SPP resin showed significantly higher values than the other groups for Tc, according to the post-curing time (p < 0.01). The Δa values denoted that the green coordinate was more present as the post-curing time increased for the SPP groups at both exposure times per layer. The PZP Tm 5- and 10-minute and SPP Tc 10- and 15-minute groups showed significant differences in comparison with the other groups (p < 0.001) when evaluated at the same exposure time. When the exposure times per layer were compared, significant differences were observed between the groups (p < 0.001). For Δb values, the yellow coordinate was more present in the resins of the PZP group and the blue coordinate in the SPP group. The SPP groups showed a significant difference in comparison with the PZP groups when analyzed individually for each exposure time per layer (p < 0.01). Moreover, significant differences were observed as the post-curing time increased for the SPP Tm 15-minute group in comparison with the SPP Tm 5- and 10-min groups (p < 0.001), and for the SPP Tc 10- and 15-min groups (p < 0.01) in comparison with the SPP Tc 5-min group. Only the SPP resin showed significant differences when the exposure time per layer was evaluated at all post-curing times (p < 0.05).

### Cell cytotoxicity

**Figure 6:**
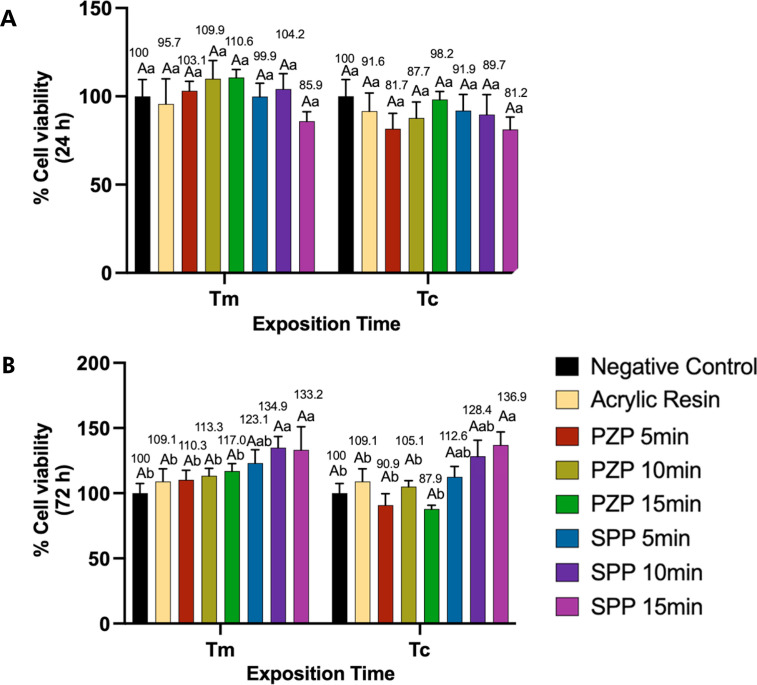
MTT assay. Bars represent the mean and standard deviation for cell viability at 24 (a) and 72 hours (b). Capital letters allow comparison between Tm and Tc for each group.

The MTT test for NOK-Si cells corresponded to the average cell metabolism values for the times analyzed (24 and 72 h), as shown in Figure 6. After 24 h, the analyzed resins were cytocompatible, regardless of the post-curing and exposure times. No significant reduction in cell viability was observed during the period analyzed (p > 0.05). At the 72-h time-point, no significant reduction in cell viability values occurred for any of the experimental groups in comparison with the negative control group (p > 0.05). Furthermore, when the SPP resin was post-cured for 10 or 15 min, a significant increase in cell viability was observed in comparison with the control for Tm (p < 0.05), and the same effect was observed with the 15-min post-curing time for Tc (p < 0.05).

Lowercase letters allow comparison between groups at each exposition time. Different letters indicate statistically significant differences (One-way ANOVA/ Tukey’s test. p < 0.05).

### Residual monomer leaching:

**Figure 7:**
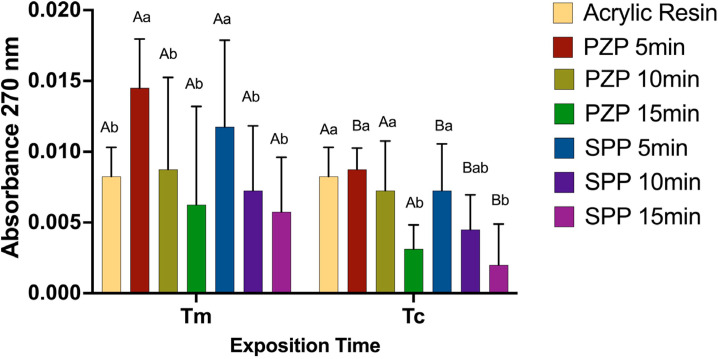
Residual monomers leaching. Bars represent mean and standard deviation. Capital letters allow comparison between Tm and Tc for each group.

The absorbance results of the extracts at 270 nm over 24 h are shown in Figure 7. In general, monomer leaching occurred in all experimental groups. For the 3D-printed resins, this leaching tended to be inversely proportional to the post-curing and exposure times per layer. For the PZP resin, a significant reduction in leaching was observed in comparison with the acrylic resin for the 15-min post-curing time for Tm (p < 0.001); however, an intense increase in leaching was observed for the 5-min post-curing time for Tm in comparison with that for Tc. For the SPP resin, significantly lower values than those of the acrylic resin were observed for Tc only for the 15-min post-curing time (p < 0.001); this difference was not observed for Tm (p > 0.05). Similar to PZP, a significant increase in the leaching of SPP resins occurred for Tm in comparison with Tc when the post-curing times were 5 and 10 min (p < 0.001).

Lowercase letters allow comparison between groups at each exposition time. Different letters indicate statistically significant differences (One-way ANOVA/ Tukey’s test. p < 0.05).

## Discussion

The digital workflow involves acquiring and processing data and manufacturing parts.^8,15^ For the 3D-printing manufacturing process, Nulty^16^ (2022) showed that digital light processing (DLP), stereolithography (SLA), and masked SLA (MSLA) printing technologies have similar accuracy and precision. However, other studies have indicated that the layer thickness, inclination of the printed part, exposure time of the layer to polymerization, and post-curing protocol can interfere with the accuracy, trueness and quality of the printed part.^1,5^ A layer thickness of up to 100 μm provides a high standard of detail for printing dental parts, such as models and provisional restorations, and printing parts at 90 degrees is more accurate than at other angles (0, 15, and 45 degrees).^1,5,19^

Calibration of the resin exposure time is recommended to ensure consistent and accurate printing and should be implemented for each printer and type/brand of resin to be used.^7,8^ This process involves 3D printing of a device that is then measured and compared to the CAD project specifications. Any geometry with known dimensions can be used for calibration. In our project, we opted to use a calibration tool provided by a resin manufacturer (Makertech Labs) because its geometry suited our requirements. However, any other calibration tool can be used without compromising the measurement accuracy. Therefore, in the first stage of this study, two exposure times were established: one indicated by the manufacturer for use with the 3D printer, and the other determined with the calibrator (Tm and Tc, respectively). For the post-processing of the printed objects, cleaning was performed according to the manufacturer’s recommendations, and three post-curing times were tested (5, 10, and 15 min).

A print trueness test was conducted to determine the effects of the selected parameters on the feasibility of the 3D printing process. The resolution of a 3D printer must be defined on each of the x-, y-, and z-axes in micrometers or dots per inch, where the z-axis corresponds to the thickness of the layer.^16^ Trueness refers to the ability of a 3D printer to manufacture the same object with consistent dimensions, and veracity refers to the discrepancy between the printed object and the actual dimensions of the desired object.^8,20,21^ The results of this study indicated that the exposure time determined by the calibrator provided greater trueness for the impression process, with the resin showing volumetric expansion as the exposure time increased. This effect was material-dependent because Tc was more effective for the PZP group, showing significantly greater trueness than Tm in both the x- and y-axes. For the SPP resin, the post-curing time also affected the trueness, with a longer time (15 min) yielding values closer to the ideal. This effect may be related to the polymerization kinetics of the 3D-printed resins. The formation of a polymeric network with a denser structure can result in volumetric contraction or shrinkage. The polymerization of the resin directly affects these parameters.^22-24^

Another important physical parameter tested in this study was the surface roughness of the printed resins, since this parameter influences biofilm adhesion, monomer leaching, and color stability.^19,25-27^ The results of this study indicated that both 3D-printed resins processed on the basis of all the parameters showed lower surface roughness (Ra) values than the acrylic resin. The exposure time per layer had a significant effect only on the PZP resin, wherein the surface roughness was reduced when processing the print with Tc in comparison to that with Tm. For the SPP resin, this effect was not observed at post-curing times of 10 and 15 min, resulting in a significant reduction in the roughness of Tm, with a less intense effect on Tc. This result indicates that the difference in the composition of the 3D-printed material is an important factor affecting the surface smoothness, and that the printing process also affects this parameter.

In this study, the surface roughness was assessed immediately after post-processing without any surface polishing. As a result, the resins had high roughness values, above 0.2 μm, which is considered the limit for plaque accumulation.^28,29^A previous study had demonstrated that an Ra value between 0.2 and 0.3 μm is achieved after polishing 3D printed provisional resins, which was maintained even after immersion in a humid environment for periods of up to 7 days,^6^ and after simulated brushing and artificial aging,^25^ whereas acrylic resins showed a tendency toward increased roughness.

The color stability was more strongly influenced by the type of resin, according to the different processes. The PZP resin showed no significant variations in ΔE*_ab_ and ΔE_00_, regardless of exposure or post-curing time; similar findings were obtained for all the other color parameters tested (ΔL, Δa, Δb). However, the SPP resin showed a significant increase in the ΔE*_ab_ and ΔE_00_ values when printed on Tc in comparison with Tm, depending on the post-curing time. Furthermore, the increments in ΔE*_ab_ and ΔE_00_ were proportional to the post-curing time, demonstrating that the polymerization process highly influences this resin. In the assessments of perceptibility, all groups showed ΔE_00_ values above the threshold (1.01).^30^ Nevertheless, ΔE_00_ values above the acceptability threshold (2.66)^30^ were mostly influenced by the Tc and SPP resins, in which ΔE_00_ values varying from 1.9 to 5.6 times above the threshold were recorded. The color change observed for SPP was associated with an increase in ΔL and a reduction in the Δa and Δb values at Tc in proportion to the post-curing time. Thus, the luminosity of this resin increased and the resin became clearer when the post-curing time was extended. Nevertheless, the whiteness index adapted for dentistry, which has been considered the ideal calculation to observe the whiteness effect,^31^ was not calculated. This can be considered as a limitation of this study.

The color stability of 3D-printed resins is a controversial parameter. This may be due to the different brands of resin tested in different studies, which may present similar^25^ or lower stability^32,33^ than chemically activated acrylic resins. In addition to the composition, the surface polishing procedure can also influence this parameter.^25^ The literature indicates that the low color stability of 3D-printed resins may be related to the high hydrophilicity/polarity of the polymers, the absence of filler particles, the presence of residual monomers, and high solubility, depending on the material and the post-curing protocol.^32-35^ The findings of these previous investigations support the results of the present study.

Few studies have evaluated the cytotoxicity of 3D-printed materials for provisional restorations.^7,22,36^ The cytotoxicity of 3D-printed resins was evaluated against a lineage of human oral keratinocytes using a cell viability assay, in which the cells were exposed to extracts of the printed resins as per the ISO 10993-5 standards.^7,17,22^ Previous studies demonstrated that in the absence of post-curing, printed provisional resins reduced cell viability by more than 80%.^9^ However, the combination of post-curing chamber power and post-curing time had a bearing on the degree of conversion of 3D-printed resins, eliminating cell cytotoxicity.^9,37,38^ The L929 fibroblast cell line was used in the aforementioned studies and the ISO 10993-5:2009 standards were followed, similar to the present study.

In a study performed by Alamo et al.^6^ (2022), oral keratinocytes were maintained in close contact with the surface of 3D-printed resins in an organotypic model, and a considerable reduction in cell viability was observed when a post-curing time of only 1 min was used. As reported previously, when the manufacturer’s protocol was used, no cytotoxicity was observed.^6^ The extent of residual monomer leaching has been reported to be correlated with the degree of conversion.^9,37,38^ Therefore, the leaching of residual monomers in our study may have been a consequence of the lower degree of conversion when layer exposure and post-cure times were reduced. However, the absence of this analysis may be considered a limitation of the present study.

Our results highlighted the importance of establishing a suitable exposure time per layer and post-curing processing for promoting 3D printing of temporary materials with improved trueness, ensuring adequate surface roughness and color stability, and minimizing residual monomer leaching. Therefore, the null hypothesis was rejected. Nevertheless, these results reveal that these parameters are also resin-dependent, indicating the need for further studies to understand the ideal formulation of 3D-printed provisional resins. Importantly, this was an in vitro study with inherent limitations. Only one printer and UV chamber were used to prepare the specimens. Color analysis was performed using the equipment indicated for tooth color measurement; however, color stability over time was not considered. Biological analyses were also performed using the resin extracts. Direct-contact experiments using more realistic models would improve our understanding of the biological compatibility of this new class of resins. Therefore, further experiments using 3D-printed resins indicated for dentistry are important to understand their behavior under the challenges found in the oral cavity and to emulate their clinical use.

## Conclusion

Changing the exposure time per layer for calibrating the 3D-printed resins positively affected the trueness of the PZP resin in both axes (x and y) without causing major changes in the other parameters investigated. The post-curing time positively affected all the properties evaluated. In addition, the post-curing time did not affect the biological compatibility of the resins. All the materials were cytocompatible with oral epithelial cells.
